# 임부의 분만 자신감, 산전 우울, 분만 지식과 배우자 지지는 분만 두려움에 영향을 미치는가?

**DOI:** 10.4069/kjwhn.2020.12.14

**Published:** 2020-12-21

**Authors:** Hyunjin Cho, Sukhee Ahn

**Affiliations:** College of Nursing, Chungnam National University, Daejeon, Korea; 충남대학교 간호대학

**Keywords:** Childbirth, Confidence, Depression, Fear, Pregnancy, 출산, 자신감, 우울, 두려움, 임신

## Abstract

**Purpose:**

This study investigated levels of childbirth fear and related prenatal factors (self-confidence for childbirth, prenatal depression, knowledge about childbirth, and spousal support) among pregnant women in South Korea.

**Methods:**

A correlational study design was used to explore levels of childbirth fear and related prenatal factors in 200 pregnant women over 28 weeks of gestation. A self-administered questionnaire was used to measure fear of childbirth and related factors, such as self-confidence for childbirth, prenatal depression, knowledge about childbirth, and spousal support.

**Results:**

One-third of the pregnant women were aged 35 years and older. Sixty-one percent of women were nulliparae, but only 26.0% had experienced prenatal education. The mean score for fear of childbirth was 66.99 out of 165. The prevalence of fear of childbirth was 72.0%, and childbirth fear was severe in 26.5% of the participants and moderate in 45.5%. Fear of childbirth was negatively related to self-confidence (r=-.45, *p*<.001), but positively related to prenatal depression (r=.21, *p*=.002). Two significant predictors were found to explain the fear of childbirth. Higher self-confidence for childbirth was associated with less severe fear of childbirth (β=–.44, *p*<.001), while higher prenatal depression was associated with more severe fear of childbirth (β=.13, *p*=.038).

**Conclusion:**

The level of fear of childbirth was higher among pregnant women with lower self-confidence and higher prenatal depression. Reasonable evidence should be provided for implementing prenatal and childbirth classes to reduce pregnant women’s depression and to increase their confidence.

## Introduction

### 연구 필요성

임신과 분만은 여성 생애주기의 중요한 사건 중 하나로 특히 분만은 여성에게 여러 신체적 경험뿐 아니라 정서적 스트레스도 동반한다[1]. 여성들에게 임신과 분만은 기쁨과 성취에서 불안과 공포에 이르기까지 모든 종류의 다차원적 정서적 경험과 감정을 유발하는데, 일부 임산부들은 신체적 변화와 호르몬의 변화 등에서 오는 부정적인 감정이 지속적으로 이어져 분만에 대한 두려움을 경험한다[2]. 분만 두려움(fear of childbirth)은 임부들에게 흔한 감정이지만, 임신 중 불안 장애 또는 심각한 두려움의 형태로 발전할 수 있기 때문에 심각한 문제이다[3].

분만 두려움의 유병률은 20%로, 그 중 약 5%에서 10%의 여성들이 심각한 수준의 분만 두려움을 보인다[4]. 심각한 분만 두려움은 ‘tocophobia’로도 일컬어지며[1], 메타 분석에서 유병률은 14%로 나타났다[3]. 분만 두려움은 정도에 따라서 낮은 수준, 보통 수준, 심각한 수준으로 구분된다. 낮은 수준과 보통 수준의 두려움은 ‘임부의 일상생활을 방해하지 않는 정도의 불안’을 말하나, 심각한 수준의 두려움은 ‘임부의 일상 기능과 복지에 장애를 일으킨 강한 불안’으로 정의된다[4]. 분만 두려움을 겪는 임부는 밤낮 항상 긴장된 상태로 불안, 불안정한 모습을 보이며, 직장과 가정에서 악몽, 집중력 부족, 괴롭고 고통스러운 생각의 반복과 피로 등의 증상들을 호소한다[2]. 심각한 분만 두려움을 겪는 임부들은 신체적, 심리적으로 부정적인 증상뿐 아니라 조기 진통이나 재태기간의 단축과 같은 문제까지 경험한다[5]. 분만 두려움은 분만 형태에도 영향을 미치며, 분만 두려움이 높은 임부일수록 질 분만보다 제왕절개 분만을 선택하는 비율이 높게 나타났다[3,6].

최근 연구에서 심각한 분만 두려움은 부정적 출산 결과를 가져올 뿐만 아니라 산후 외상 후 스트레스 장애(post-traumatic stress disorder)와도 관련이 있음을 보고하고 있으며[7], 분만 두려움은 임신 전후 우울과 연관성을 보인다[8]. 이러한 점들로 미루어 볼 때, 분만 두려움의 발생에 영향을 주는 산전 관련요인들을 탐색하는 것은 분만 두려움을 극복함으로써 산후 긍정적인 출산 경험을 성취하고, 부모로서의 적응 및 가족의 건강을 달성하도록 돕는 간호 중재의 수립에 중요한 자료가 될 것이다.

문헌고찰을 통해 분만 두려움은 분만 자신감, 산전 우울, 분만과 관련된 지식 및 배우자 지지와 관련이 있음을 확인하였다. 분만 자신감은 분만 두려움을 낮추는 예측 요소로 분만 두려움이 클수록 분만 자신감은 낮아진다[6]. ‘분만과정에 대한 무지’와 ‘스스로 통제할 수 없으나 겪어야만 하는 과정’이라는 인식은 분만 두려움을 갖게 하며, 그 결과 분만에 대한 자기 대처 능력의 저하로 분만 자신감을 감소시키고 분만 두려움을 더욱 증가시키게 된다[9]. 산전 우울은 임신 중 분만 두려움을 심화시킬 수 있으며[10], 우울할 때 분만 두려움이 발생할 승산은 우울이 없는 임부보다 4.8배 높았다[11]. 분만 두려움은 또한 아직 발견되지 못한 산전 우울의 징후일 수 있다[8]. 임부의 분만 두려움이 높을수록 산후 우울의 발생 위험도가 높게 나타난 결과는 분만 두려움이 산후 우울과 관련 있음을 시사한다[12]. 반면 분만과 관련된 교육은 분만 두려움을 감소시키며, 분만과 관련된 지식이 많을수록 임부의 분만 자신감을 크게 높이고 분만 두려움을 완화시킨다[6]. 긍정적인 배우자 지지는 임부에게 분만 자신감을 증진시키고, 분만 시 통증, 상태 불안, 스트레스를 감소시킨다[13]. 또한 배우자 지지를 충분히 받은 임부는 분만을 긍정적으로 수용한 결과[14]를 볼 때, 배우자 지지는 분만 두려움을 감소시킬 수 있는 요인으로 예상할 수 있다.

임부의 산과력 지표 중에서 출산 경험에 따른 분만 두려움의 차이 유무는 연구결과가 아직 혼재되어 있다. 유럽이나 호주 연구에서는 초산모와 경산모 사이에 분만 두려움의 차이가 없다고 보고한[4,7] 반면, 스웨덴 연구에서는 초산모가 경산모보다 더 높은 분만 두려움을 가진다고 보고하였다[15,16]. 출산 선호도와 분만 두려움의 관계 연구에 따르면, 분만 두려움이 높은 임부는 질 분만보다 제왕절개 분만을 4.6배 선호하였고, 실제 분만에서 질 분만 대비 제왕절개 분만을 2.4배 더 선택하였다[17]. 분만 경험에 따른 비교연구에서 분만 두려움이 있는 초산모가 제왕절개 분만을 선택하는 정도는 질 분만을 선택하는 경우보다 11.79배 더 높았고, 경산모의 경우 제왕절개 분만을 선택하는 경우가 질 분만을 선택하는 경우보다 8.32배 더 높게 나타났다[16]. 분만 두려움에 대한 연구는 주로 북부 유럽, 북미 및 호주의 임산부들을 대상으로 활발하게 이루어져 왔고[3,7,9] 분만 두려움에 대한 선행 요인과 결과에 대한 요인 탐색도 국외 연구에서 주로 찾을 수 있다[2,6,16,18,19]. 반면 국내 연구는 임부의 태교, 불안, 분만 통증과 임신기 스트레스[11,20]에 대한 횡단성 조사 연구가 대부분으로, 분만 두려움 수준을 확인하고 관련요인을 탐색하는 연구는 찾기 어렵다. 이에 본 연구는 임부를 대상으로 분만 두려움 수준을 확인하고, 분만 두려움에 산전 관련요인(산과력, 분만 경험, 분만 자신감, 산전 우울, 분만에 대한 지식, 배우자 지지)의 영향력을 탐색하여 이를 통해 추후 임산부 교육을 기획하거나 출산 준비 교육 내용의 타당성에 근거를 제공하고자 한다.

### 연구 목적

본 연구는 임부의 분만 두려움 수준을 확인하고 분만 두려움에 영향을 미치는 산전 관련요인을 탐색하기 위함이며, 구체적인 연구의 목적은 다음과 같다.

• 임부의 분만 두려움 수준과 산전 관련요인(분만 자신감, 산전 우울, 분만에 대한 지식, 배우자 지지) 수준을 확인한다.

• 임부의 일반적 특성과 산과적 특성에 따른 분만 두려움의 차이를 확인한다.

• 임부의 분만 두려움과 산전 관련요인(분만 자신감, 산전 우울, 분만에 대한 지식, 배우자 지지) 간 관계를 확인한다.

• 임부의 분만 두려움에 영향을 미치는 산전 관련요인을 탐색한다.

## Methods

Ethics statement: This study was approved by the Institutional Review Board of Chungnam National University (201904-SB-0440-01). Informed written consent was obtained from the participants.

### 연구 설계

본 연구는 임부의 분만 두려움 수준을 확인하고 산전 관련요인과의 관련성을 탐색하고자 상관성 조사 연구설계를 사용하였다.

### 연구 대상

연구대상자는 대전 지역에 소재한 2개의 여성 전문병원에서 규칙적인 산전 관리를 받는 임부이다. 대상자 선정기준은 임신 28주 이상으로 산과적 합병증이 없으며, 건강한 단태아를 가진 임부이다. 제외 기준에는 임신 전부터 앓고 있는 정신건강 문제가 있는 경우, 모체 또는 태아의 건강 문제로 37주 이전에 조산을 계획하는 경우이다. 임부를 28주 이상으로 선정한 이유는 임신 28주 이상에서 임신 상태 및 불안으로 분만 두려움의 승산비를 2.7배 증가시키는 결과[21]가 보고되었기 때문이다. 본 연구에 필요한 표본 크기는 표본크기를 산정하는 G*power 프로그램을 사용하여 유의수준(α)=0.05, 검정력(1–β)=0.8로 설정하였다. 선행연구에서 보고한 분만 두려움과 출산에 대한 자기효능감 관계가 초산부에서 r=.37, 경산부에서 r=.36에 근거하여 상관관계 r=.3으로 추정하였고[22], 이를 회귀분석에서 필요한 효과 크기로 계산한 결과 f²=0.10이었다. 본 연구의 회귀식에 필요한 독립변수 6개를 입력할 때 필요한 표본 크기는 최소 143명으로 나타났다. 이에 설문지 수거율 및 부정확한 설문지 탈락을 고려하여 임부 230명을 모집하였고, 자료 입력 시 응답이 미비한 30명의 자료는 제외한 결과 최종 연구대상자는 200명이었다.

### 연구 도구

#### 분만 두려움

본 연구에서는 Wijma 등[23]이 임신 기간 중과 출산 후의 분만 두려움을 측정하기 위해 만든 W-DEQ (Wijma Delivery Expectancy/Experience Questionnaire) 도구를 한국어로 번역한 도구를 사용하였다[24]. 본 연구에서는 산전 분만 두려움과 분만에 대한 기대감을 평가하는 도구인 버전 A를 사용하였다. 분만 두려움 버전 A는 33문항으로, 0–5점으로 구성된 리커트 척도이며, 14개 문항(2, 3, 6, 7, 8, 11, 12, 15, 19, 20, 24, 25, 27, 31번 문항)은 역채점하고 총점은 0에서 165점의 범위를 갖는다. 점수가 높을수록 분만 두려움이 높음을 의미한다. 분만 두려움 수준에 따라 분만 두려움 점수의 합이 65점 미만인 경우 낮은 두려움, 65이상 85점 미만은 보통의 분만 두려움, 85점 이상은 심각한 분만 두려움으로 나눌 수 있다. 원 도구의 신뢰도는 Cronbach’α=.89, 한국판 도구의 평가 연구에서 신뢰도는 Cronbach’α=.89, 본 연구의 신뢰도는 Cronbach’α=.85였다.

#### 분만 자신감

본 연구에서는 Lee [25]가 개발한 분만 자신감 척도를 이용하여 15문항 4점 척도로 측정하였다. 각 문항에 대한 응답은 ‘전혀 그렇지 않다’ 1점, ‘조금 그렇다’ 2점, ‘보통 그렇다’ 3점, ‘대단히 그렇다’ 4점으로 점수가 높을수록 분만 자신감이 높음을 의미한다. 개발 당시 도구의 신뢰도는 Cronbach’s α=.89였고, 본 연구에서는 Cronbach’s α=.82였다.

#### 산전 우울

산전 우울은 Cox 등[26]이 개발한 Edinburgh Postnatal Depression Scale (EPDS)를 Kim 등 [27]이 번역한 한국어판을 사용하여 측정하였다. EPDS는 임신 중 우울 측정에도 타당도를 인정받아 널리 쓰이고 있다. 우울, 불안 및 공포, 죄책감, 자해사고 등에 대해 자가보고 형식의 10문항으로 구성되어 총 점수 범위는 0–30점까지이다. 0–3점까지의 4점 척도로 1, 2, 4번 문항을 제외한 나머지 문항은 역채점하고, 점수가 높을수록 우울한 것으로 평가한다. 한국판 EPDS의 최적 절단점을 9/10점으로 평가하여 해당 절단점을 기준으로 본 연구에서 우울의 정도를 나누었다[27]. 개발 당시 도구의 신뢰도를 Cronbach’s α=.87로 제시하였고, 한국판 우울 도구의 신뢰도는 Cronbach’s α=.84, 본 연구에서는 Cronbach’s α=.86이었다.

#### 분만에 대한 지식

분만에 대한 지식은 Choi [28]가 분만생리, 분만과정, 산전운동, 호흡법 및 힘주는 법에 관련된 지식수준을 묻는 20개의 문항으로 개발한 도구를 사용하여 측정하였다. ‘예’, ‘아니오’로 답하게 한 후 정답은 1점, 오답은 0점으로 처리하였다. 점수의 범위는 0점에서 20점으로 점수가 높을수록 분만에 대한 지식이 많음을 의미한다[28].

#### 배우자 지지

배우자 지지는 Kessler [29]가 개발한 도구를 Jang [13]이 번안한 도구를 사용하여 측정하였다. ‘배우자는 나에게 관심을 기울인다’, ‘나에게 심각한 문제가 생기면 나는 배우자에게 의지한다’, ‘나는 배우자와 함께 있으면 편안하다’와 같은 6문항으로 구성되어 있다. 각 문항은 ‘전혀 그렇지 않다’ 1점에서 ‘정말 그렇다’ 4점까지이며, 점수 범위는 최소 6점에서 최고 24점으로 점수가 높을수록 지지도가 높다. 원 도구의 신뢰도는 Cronbach’s α=.84, 한국판 도구의 평가 연구에서 신뢰도는 Cronbach’s α= .91, 본 연구의 신뢰도는 Cronbach’s α= .83이었다.

#### 임부의 일반적 특성과 산과적 특성

임부의 일반적 특성은 임부의 나이, 학력, 직업, 경제소득 항목으로 임부의 산과적 특성은 출산 경험, 산전 교육 경험, 계획 임신으로 구성되어 있다. 일반적 특성에서 나이, 학력, 직업은 주관식으로 자필 응답하도록 하였고 경제소득은 ‘400만 원 미만, 400만 원 이상’으로 선택 응답하도록 하였다. 산과적 특성으로 ‘출산 경험, 산전 교육, 계획 임신 여부, 출산 방식 선호도’를 조사하였다. 출산 방식 선호도는 임부가 분만 시 선호하는 방법으로 ‘질 분만’과 ‘제왕절개 분만’ 중 원하는 방식을 선택 응답하도록 하였다.

### 자료 수집

제1연구자는 연구 도구에 대해 원 개발자에게 사용 승낙을 받았다. 자료 수집은 생명윤리위원회의 승인 직후 2019년 6월 16일부터 2020년 2월 1일까지 시행하였다. 연구자는 대전 지역에 소재한 2개의 여성전문병원의 병원장과 간호부장에게 사전에 연구의 목적과 방법을 설명하고 연구 진행에 대한 허락을 구했다. 연구의 목적과 방법에 대한 설명을 듣고 연구 참여에 동의한 임부들에게 분만 두려움 수준과 산전 관련요인 설문지 및 임부의 일반적 특성과 산과적 특성에 대한 설문 조사지를 자가 작성하게 하였다. 설문 소요 시간은 10분에서 15분 내외였으며, 설문지 작성을 완료한 대상자에게 소정의 답례품을 제공하였다.

### 자료 분석

수집된 자료는 IBM SPSS for Windows, ver. 24.0 (IBM Corp., Armonk, NY, USA)을 이용하여 분석하였으며, 통계적 유의수준은 5% 미만으로 하였다.

1) 대상자의 일반적/산과적 특성, 분만 두려움, 분만 두려움 산전 관련요인(분만 자신감, 산전 우울, 분만에 대한 지식, 배우자 지지) 수준은 빈도분석과 기술통계를 이용하여 분석하였다.

2) 대상자 일반적/산과적 특성에 따라 분만 두려움에 대한 차이 검정은 t-test와 카이제곱 검정으로 분석하였다.

3) 분만 두려움과 산전 관련요인(분만 자신감, 산전 우울, 분만에 대한 지식, 배우자 지지) 간 관계는 Pearson 상관계수를 이용하여 상관관계를 분석하였다.

4) 산전 관련요인(분만 자신감, 산전 우울, 분만에 대한 지식, 배우자 지지)이 분만 두려움에 미치는 영향을 다중 회귀분석을 통해 분석하였다.

## Results

### 대상자의 일반적 특성과 산과적 특성

임부의 나이는 35세 미만이 135명(67.5%), 35세 이상은 65명(32.5%)이었다. 직업이 없는 경우는 111명(55.5%)이었고, 대학교 졸업 이상이 185명(92.5%), 수입이 400만 원 이상인 경우가 115명(57.5%)으로 나타났다. 임부의 산과적 특성에서는 임부의 과반수 이상(61.0%)이 처음 출산을 기대하고 있고, 경산부(출산 1회 이상)가 78명(39.0%)이었다. 계획된 임신을 한 여성은 135명(67.5%), 산전 교육을 경험한 여성은 52명(26.0%)이었다. 선호하는 출산 방식은 질 분만이 180명(90.0%), 제왕절개 분만이 20명(10.0%)이었다(Table 1).

### 임부의 분만 두려움 수준과 산전 관련요인 수준

임부의 분만 두려움의 평균점수는 66.99±1.25점이고, 최소 12점에서 최대 140점을 보였다. 분만 두려움 수준을 세 단계로 나누어 평가한 결과, 65점 미만의 낮은 분만 두려움을 경험하는 여성은 56명(28.0%), 65점에서 84점의 보통의 분만 두려움을 경험하는 여성은 91명(45.5%), 85점 이상의 심각한 분만 두려움을 경험하는 여성은 53명(26.5%)으로 나타났다. 보통의 분만 두려움을 보고한 비율이 가장 많았고, 다음으로 낮은 분만 두려움, 심각한 분만 두려움 순이었다.

산전 관련요인으로 분만 자신감, 산전 우울, 출산에 대한 지식, 배우자 지지의 수준을 확인하였다. 분만 자신감은 21점에서 60점의 범위를 보이며, 평균 41.46±9.01점으로 중간 이상 수준으로 나타났다. 산전 우울은 0점에서 27점의 범위를 보였고, 평균 8.60±5.19점으로 낮은 수준으로 나타났다. 산전 우울은 총점 10점을 기준으로 ‘우울이 낮은 임부’와 ‘위험이 있는 임부’의 경우로 나누어 평가한 결과, 우울감이 낮은 임부는 133명(66.5%), 10점 이상으로 우울 위험이 있는 임부는 67명(33.5%)으로 나타났다. 분만에 대한 지식은 13점에서 22점의 분포를 보이며, 평균 13.22±1.55점으로 중간 이상 수준이었다. 배우자 지지는 최소 7점에서 최대 24점이었고, 평균은 20.96±3.06점으로 높은 수준이었다(Table 2).

### 임부의 일반적 특성과 산과적 특성에 따른 분만 두려움 차이

임부의 나이, 직업, 학력, 경제상태에 따른 분만 두려움의 차이를 확인한 결과, 경제상태를 제외한 일반적 특성에는 유의한 차이가 없었다. 경제상태에서는 400만 원 미만 월수입이 있는 임부에서 400만 원 이상인 임부보다 분만 두려움 점수가 유의하게 높았다(t=2.58, *p*=.010). 산과적 특성 중 계획 임신 여부에 따라 분만두려움에 차이가 있었다(t=-2.35, *p*=.019). 임신계획을 한 임부는 임신을 계획하지 않은 임부에 비해 분만두려움 점수가 높았다. 반면 분만 경험, 계획 임신 여부, 산전 교육 유무와 출산 방식 선호도에 따라 분만 두려움은 유의한 차이를 보이지 않았다(Table 1).

### 분만 두려움과 산전 관련요인 간 관계

분만 두려움은 분만 자신감(r=–.45, *p*<.001)과 중간 수준의 유의한 음의 상관관계를 보였고, 산전 우울(r=.21, *p*=.002)과 낮은 수준의 유의한 양의 상관관계를 보였다. 산전 관련요인 간 관련성을 살펴보면, 분만 자신감은 산전 우울(r=–.20, *p*=.004)과 유의한 음의 상관관계를 가졌고, 분만 자신감은 배우자 지지(r=.27, *p*<.001)와 유의한 양의 상관관계를 보였다. 산전 우울은 배우자 지지(r=–.15, *p*=.030)와 유의한 음의 상관관계를 보였다. 분만 지식은 분만 자신감(r=.15, *p*=.033)과 유의한 양의 상관관계를 보였다(Table 3).

### 분만 두려움에 영향을 미치는 산전 관련요인 탐색

다중 회귀분석을 위해 분만 두려움을 종속변수로 입력하였고, 산전 관련요인 4개(분만 자신감, 산전 우울, 분만에 대한 지식, 배우자 지지)는 독립변수로 입력하였다. 독립변수들의 다중 공선성을 공차한계와 분산팽창계수(variance inflation factor)로 확인한 결과, 공차 한계 값은 0.87–0.99, 분산팽창계수 값은 1.02–1.14의 범위에 있어 다중공선성의 문제가 없는 것으로 나타났다. Durbin-Watson 값이 2.08로 기준 2에 가까워 오차의 독립성을 확인하였다. 다중 회귀식을 검정한 결과 회귀식은 유의하였고(F=14.07, *p*<.001), 4개 독립변수에 대한 설명력은 20.8%였다. 4개 입력변수 중에서 유의한 설명변수는 분만 자신감(t=–6.49, *p*<.001)과 산전 우울(t=2.08, *p*=.038)로 나타났다. 영향력 크기를 비교해보면 분만 자신감(β=–.44)이 산전 우울(β=.13)보다 영향력이 크다. 즉 임부의 분만 두려움은 분만 자신감이 낮을수록, 산전 우울이 높을수록 높아지는 것으로 나타났다(Table 4).

## Discussion

본 연구에서 임부의 분만 두려움은 165점 만점에 66.99점이었고, 분만 두려움 발생률은 87.5%, 그 중 심각한 분만 두려움 발생률은 29.0%로 나타났다. 국내에서는 처음 산전 분만 두려움을 평가한 것이라 국내 임부의 수준과 비교할 자료가 없는 실정으로 국외 연구와 비교하고자 한다. 유럽 임부를 대상으로 한 분만 두려움 연구[30]에서 ‘보통 두려움’의 점수와 ‘심각한 두려움’의 비율을 살펴보면, 벨기에 55.8 (4.5%), 아이슬란드 51.4 (9.6%), 덴마크 57.8 (9.4%), 에스토니아 61.3 (15.6%), 노르웨이 60.6 (12.7%), 스웨덴 63.4 (14.6%)였다. 이와 비교 시 우리나라 임부의 점수와 발생률이 더 높게 나타났다. 이런 발생률의 차이는 연령, 교육 및 임신 연령 등 사회, 문화적 차이가 있고 조산사 및 의료 시스템의 제공 정도에 따라 국가마다, 산모마다 인지하는 수준이 다르기 때문으로 보인다. 특히 서구 국가와 달리 국내 임부에게는 표준 산전 교육이 부족하기 때문일 수 있다. 한국의 문화에서 산전 교육은 출산에 대한 두려움 해소보다는 출산 후 육아와 신생아 관리, 모유 수유 등에 집중되어 있기 때문으로 보인다. 분만 전에 두려움, 스트레스, 분만 자신감과 관련된 산전 교육을 받은 국내 임산부들 사이에서 분만에 대한 두려움이 줄어든다는 연구결과가 이 가설을 뒷받침하고 있다[16]. 따라서 추후 연구에서는 우리나라 임부의 분만 두려움을 줄이는 데 산전 교육 경험이 관련이 있는지, 산전 교육이 분만 두려움을 감소시키는 효과가 있는지 탐색할 필요가 있다.

본 연구에서 임부의 분만 자신감은 분만 두려움에 유의한 영향을 주는 것으로 나타났다. 이는 분만 자신감이 높을수록 분만 두려움이 낮아지는 선행 연구와 일치한다[26]. 분만 자신감이 낮은 임부는 분만 두려움이 높고, 분만과정에서 더 큰 통증과 스트레스를 동반한다. 분만 자신감 증진은 임부의 분만 두려움 감소에 중요한 의미를 가지기 때문에 임부의 분만 자신감 정도를 확인하고, 분만 자신감이 부족한 임부들에게 분만과정에 대한 정확한 정보와 호흡법, 이완법, 연상법 같은 분만상황에 경험하는 통증과 스트레스에 직접 대처할 방법을 교육하여 분만 자신감을 증진하는 효과적인 중재가 필요하다고 생각한다.

본 연구에서 산전 우울은 분만 두려움을 증가시키는 영향요인이었다. 선행연구에서 산전 우울은 임신 중 분만 두려움을 심화시키고[10], 우울이 있을 때 분만 두려움이 발생하는 승산비는 4.8배 [11] 더 높다는 것과 일치한다. 본 연구에서 임부 200명 중 산전 우울 위험을 가진 사람이 67명(33.5%)으로, 국외 메타 분석에서 나타난 산전 우울의 유병률 23.5%–30.8% [3]와 비교하였을 때 2.7% 더 높았다. 따라서 간호사는 산전 우울을 미리 사정하고 그 결과를 바탕으로 분만 두려움의 정도를 확인하며, 임부의 자신감을 증진하고 정보를 제공하는 등 산전 우울을 감소시킬 수 있는 적절한 중재를 제공할 필요가 있다.

본 연구에서는 분만에 대한 지식은 분만 두려움과 관련성이 없는 것으로 나타나 분만에 대한 지식이 분만 두려움을 감소시킨다는 선행연구[8]와 일치하지 않았다. 본 연구에서는 대상자가 주체적으로 책이나 인터넷 등의 매체를 찾아보며 기존에 알고 있던 지식을 ‘맞다’, ‘아니다’ 형식으로 단순 응답하였기 때문에, 전문적인 산전 교육이 아닌 인터넷에서 자발적으로 얻은 전문적이지 않고 부정확한 지식은 분만 두려움을 감소시키는 데 영향을 주지 못함을 확인한 것이다. 분만에 대한 지식이 분만 두려움을 감소시킨 선행연구[8]는 교육 중재 프로그램을 통해 전문적인 정보를 제공하고 중재에 능동적으로 참여한 임부의 분만 두려움을 측정한 결과였다. 단순히 지식의 정도를 측정하는 것에서 그치지 않고 올바르지 못한 지식은 바로 고쳐주며, 분만에 대한 긍정적인 지식을 제공해 주는 신뢰할 만한 출산 준비 교육 중재가 분만 두려움을 감소시키는 데 중요함을 확인한 것이다.

본 연구에서 배우자 지지는 분만 두려움과 유의한 관련성이 나타나지 않았다. 선행연구에서는 배우자의 지지와 사회적 지원이 부족하면 분만 두려움이 증가한다는 결과를 보여주었지만[13], 본 연구에서 사용된 배우자 지지 도구는 결혼생활 중 배우자에게 의지하고 필요의 반응을 알아보는 광범위한 배우자 지지 정도를 알아보는 도구였기에 임신이라는 특수한 상황에서 배우자의 지지가 적절했는지 평가하기엔 어려움이 있었다. 이에 추후 연구에서는 배우자로서의 물리적, 정서적, 정보적, 재정적인 지지가 다차원적으로 제공되었는지 평가할 수 있는 도구를 사용할 필요가 있다.

대상자의 산과 특성 중에서 출산 경험에 따른 분만 두려움에는 차이가 없었다. 이는 유럽의 선행연구 중 초산모와 경산모 사이에 분만 두려움의 전반적인 차이를 발견하지 못한 연구[8]와 일치하였으나, 출산 경험에 따라 분만 두려움이 다르다는 선행연구[19]도 있다. 따라서 출산 경험과 관계없이 분만 두려움에 대한 사정과 중재가 필요하다. 분만 두려움이 높은 초산모의 경우 분만과정과 불확실성에 대한 두려움을 사정하고 정보 제공과 상담을 통해 불확실성을 개선하며, 경산모의 경우 이전의 분만의 경험을 사정하고 적절한 중재를 제공하기 위한 노력이 필요하다.

본 연구에서 분만 두려움은 ‘선호하는 출산 방식’에 따른 차이가 없는 것으로 나타나, 분만 두려움과 선호하는 출산 방법 사이에 통계적으로 유의한 관계가 없다는 연구결과[16]와 일치하였다. 하지만 분만 두려움이 높을수록 제왕절개 분만을 선호하는 선행연구[19]와는 차이가 있었다. 이런 결과는 분만 두려움 외 다른 요인들도 제왕절개 분만 선택에 영향을 줄 수 있기에 출산 방식 선호 관련요인을 추가로 탐색할 필요가 있다. 제왕절개 분만을 선택하는 임부들은 ‘질 분만에 대한 두려움, 아기의 안전에 대한 걱정’보다 분만과정에서 발생하는 ‘통증에 대한 두려움’ 때문에 제왕절개 분만을 선택한다고 보고하였다[16]. 이는 제왕절개의 선택에 분만 두려움만 영향을 주는 것이 아니라 통증, 분만 자신감 저하, 우울, 분만 선호도와 분만 방식의 일치 여부 등 다른 요인들도 영향을 줄 수 있기 때문이라 생각한다. 따라서 산전에 임부의 출산 방식 선호도를 확인하고 분만 두려움 수준을 평가하여 분만 두려움 수준이 높은 임부에게 두려움이 감소시키는 중재를 제공하는 것이 중요하다.

본 연구에서는 분만 자신감이 높을수록, 산전 우울이 낮을수록 분만 두려움은 낮아지는 것을 확인할 수 있었다. 이 결과를 바탕으로 임산부 산전 교실을 기획하거나 출산 정보를 제공하는 데 근거를 제공할 수 있을 것이다. 간호 실무에서는 임신 중반기를 넘은 임부를 대상으로 산전 우울과 분만 자신감 및 분만 두려움에 대한 사정이 필요하다. 간호사는 분만 두려움 수준이 심각한 임부에게는 계획된 출산 준비 교육을 참여하게 하고 산전 우울을 낮출 수 있는 자원과 방법을 안내할 필요가 있다. 이를 통해 분만 자신감을 획득하고 산전 우울은 감소시킴으로써 분만 두려움을 낮추어, 건강한 임신기에 적응하며 긍정적인 출산 경험을 할 수 있도록 도와야 할 것이다.

본 연구의 대상자는 지역적으로 한 지역에 국한되어 있고, 연구자의 편의에 의해 임의로 표출하여 선정하였기 때문에 본 연구결과를 일반화하기에는 제한이 있다. 또한 본 연구는 횡단성 조사연구로 일회성의 연구로 끝나 추후 연구가 필요하다. 임부의 산전·산후 분만 두려움 관련요인을 파악하는 것은 중요하므로 본 연구에서 탐색한 산전 관련요인(분만 자신감, 산전 우울, 분만에 대한 지식, 배우자 지지) 이외에 산후에도 보고되는 분만 두려움 관련요인을 확인하는 연구가 필요하다.

## Figures and Tables

**Table 1.** Differences in fear of childbirth according to the participants’ characteristics (N=200)

Characteristic	Categories	n (%)	Mean±SD	t	*p*
Age (year)	<35	135 (67.5)	72.38±21.17	–0.32	.287
	≥35	65 (32.5)	73.35±17.44		
Occupation	Yes	89 (44.5)	70.79±19.37	–1.20	.281
	No	111 (55.5)	74.22±20.43		
Level of education	College and above	185 (92.5)	72.22±19.63	–1.18	.125
	High school	15 (7.5)	78.60±24.01		
Monthly household income (10,000 Korean won)	<400	115 (57.5)	75.80±20.65	2.58	.010
	≥400	85 (42.5)	68.50±18.36		
Parity	Nullipara	122 (61.0)	71.61±20.18	-0.96	.720
	Multipara	78 (39.0)	74.39±19.71		
Planned pregnancy	Yes	135 (67.5)	70.41±19.76	–2.35	.019
	No	65 (32.5)	77.44±19.78		
Experience of prenatal education	Yes	52 (26.0)	71.26±22.66	–0.59	.550
	No	148 (74.0)	73.20±19.03		
Preferred delivery method	Vaginal birth	180 (90.0)	72.26±19.67	-0.91	.675
	Cesarean birth	20 (10.0)	76.60±22.91		

**Table 2.** Levels of fear of childbirth and prenatal factors (N=200)

Characteristic	Categories	Value
Fear of childbirth		66.99±1.25 (12–140)
	Low (<65)	56 (28.0)
	Moderate (65–84)	91 (45.5)
	Severe (≥85)	53 (26.5)
Self-confidence for childbirth		41.46±9.01 (21–60)
Prenatal depression	Low (<9)	133 (66.5)
	High (≥10)	67 (33.5)
Knowledge about childbirth		13.22±1.55 (13–22)
Spousal support		20.96±3.06 (7–24)

Values are presented as mean±SD (range) or n (%).

**Table 3.** Relationships among fear of childbirth and related prenatal factors (N=200)

Variable	r (*p*)			
	Fear of childbirth	Self-confidence for childbirth	Prenatal depression	Knowledge about childbirth
Self-confidence for childbirth	–.45 (<.001)	1		
Antenatal depression	.21 (.002)	–.20 (.004)	1	
Knowledge about childbirth	–.06 (.399)	.15 (.033)	–.02 (.775)	1
Spousal support	–.09 (.163)	.27 (<.001)	–.15 (.030)	–.01 (.947)

**Table 4.** Prenatal factors affecting fear of childbirth (N=200)

Variable	β	SE	t	*p*
Self-confidence for childbirth	–.44	.14	–6.49	<.001
Prenatal depression	.13	.24	2.08	.038
Knowledge about childbirth	.01	.81	0.05	.956
Spousal support	.04	.43	0.67	.504
R²=.226, adjusted R²=.208				
F (*p*)=14.07 (<.001)				
